# Temporal change in minimum mortality temperature under changing climate: A multicountry multicommunity observational study spanning 1986–2015

**DOI:** 10.1097/EE9.0000000000000334

**Published:** 2024-09-30

**Authors:** Daewon Yang, Masahiro Hashizume, Aurelio Tobías, Yasushi Honda, Dominic Roye, Jaemin Oh, Tran Ngoc Dang, Yoonhee Kim, Rosana Abrutzky, Yuming Guo, Shilu Tong, Micheline de Sousa Zanotti Stagliorio Coelho, Paulo Hilario Nascimento Saldiva, Eric Lavigne, Patricia Matus Correa, Nicolás Valdés Ortega, Samuel Osorio, Jan Kyselý, Aleš Urban, Hans Orru, Ene Indermitte, Jouni Jaakkola, Niilo Ryti, Mathilde Pascal, Veronika Huber, Alexandra Schneider, Klea Katsouyanni, Antonis Analitis, Alireza Entezari, Fatemeh Mayvaneh, Patrick Goodman, Ariana Zeka, Paola Michelozzi, Francesca de’Donato, Barrak Alahmad, Magali Hurtado Diaz, César De la Cruz Valencia, Ala Overcenco, Danny Houthuijs, Caroline Ameling, Shilpa Rao, Baltazar Nunes, Joana Madureira, Iulian Horia Holo-bâc, Noah Scovronick, Fiorella Acquaotta, Ho Kim, Whanhee Lee, Carmen Íñiguez, Bertil Forsberg, Ana Maria Vicedo-Cabrera, Martina S. Ragettli, Yue-Liang Leon Guo, Shih Chun Pan, Shanshan Li, Francesco Sera, Antonella Zanobetti, Joel Schwartz, Ben Armstrong, Antonio Gasparrini, Yeonseung Chung

**Affiliations:** a Department of Information and Statistics, Chungnam National University, Daejeon, South Korea; b Department of Global Health Policy, Graduate School of Medicine, The University of Tokyo, Tokyo, Japan; c School of Tropical Medicine and Global Health, Nagasaki University, Nagasaki, Japan; d Institute of Environmental Assessment and Water Research (IDAEA), Spanish Council for Scientific Re-search (CSIC), Barcelona, Spain; e Faculty of Health and Sport Sciences, University of Tsukuba, Tsukuba, Japan; f Climate Research Foundation (FIC), Madrid, Spain; g Spanish Consortium for Research on Epidemiology and Public Health (CIBERESP), Madrid, Spain; h Department of Mathematical Sciences, Korea Advanced Institute of Science and Technology, Daejeon, South Korea; i Faculty of Public Health, University of Medicine and Pharmacy at Ho Chi Minh City, Ho Chi Minh City, Vietnam; j Department of Global Environmental Health, Graduate School of Medicine, The University of Tokyo, Tokyo, Japan; k Universidad de Buenos Aires, Facultad de Ciencias Sociales, Instituto de Investigaciones Gino Germani, Buenos Aires, Argentina; l Department of Epidemiology and Preventive Medicine, School of Public Health and Preventive Medicine, Monash University, Melbourne, Australia; m Climate, Air Quality Research Unit, School of Public Health and Preventive Medicine, Monash University, Melbourne, Australia; n School of Public Health and Social Work, Queensland University of Technology, Brisbane, Australia; o School of Public Health and Institute of Environment and Human Health, Anhui Medical University, Hefei, China; p National Institute of Environmental Health, Chinese Center for Disease Control and Prevention, Beijing, China; q Center for Global Health, School of Public Health, Nanjing Medical University, Nanjing, China; r Department of Pathology, University of Sao Paulo School of Medicine, São Paulo, Brazil; s Institute of Advanced Studies, University of São Paulo, São Paulo, Brazil; t School of Epidemiology & Public Health, Faculty of Medicine, University of Ottawa, Ottawa, Canada; u Air Health Science Division, Health Canada, Ottawa, Canada; v Department of Public Health, Universidad de los Andes, Santiago, Chile; w Department of Environmental Health, University of São Paulo, São Paulo, Brazil; x Institute of Atmospheric Physics, Czech Academy of Sciences, Prague, Czech Republic; y Faculty of Environmental Sciences, Czech University of Life Sciences, Prague, Czech Republic; z Institute of Family Medicine and Public Health, University of Tartu, Tartu, Estonia; aa Center for Environmental and Respiratory Health Research (CERH), Research Unit of Population Health, Faculty of Medicine, University of Oulu, Oulu, Finland; bb Medical Research Center Oulu (MRC Oulu), Oulu University Hospital and University of Oulu, Oulu, Finland; cc Santé Publique France, Department of Environmental Health, French National Public Health Agency, Saint Maurice, France; dd IBE-Chair of Epidemiology, LMU Munich, Munich, Germany; ee Institute of Epidemiology, Helmholtz Zentrum München – German Research Center for Environmental Health (GmbH), Neuherberg, Germany; ff Department of Hygiene, Epidemiology and Medical Statistics, National and Kapodistrian University of Athens, Athens, Greece; gg Environmental Research Group, School of Public Health, Imperial College London, London, UK; hh Climatology Research Group, Institute of Landscape Ecology, University of Münster, Münster, Germany; ii School of Physics, Technological University Dublin, Dublin, Ireland; jj Institute of Environment, Health and Societies, Brunel University London, London, United Kingdom; kk Department of Epidemiology, Lazio Regional Health Service, ASL Roma1, Rome, Italy; ll Department of Environmental Health, Harvard T.H. Chan School of Public Health, Harvard University, Boston, Massachusetts; mm Department of Environmental Health, National Institute of Public Health, Cuernavaca, Morelos, Mexico; nn Laboratory of Management in Science and Public Health, National Agency for Public Health of the Ministry of Health, Chisinau, Republic of Moldova; oo National Institute for Public Health and the Environment (RIVM), Centre for Sustainability and Environmental Health, Bilthoven, Netherlands; pp Norwegian Institute of Public Health, Oslo, Norway; qq Department of Epidemiology, Instituto Nacional de Saúde Dr Ricardo Jorge, Porto, Portugal; rr Centro de Investigação em Saúde Pública, Escola Nacional de Saúde Pública, Universidade NOVA de Lisboa, Lisbon, Portugal; ss Department of Environmental Health, Instituto Nacional de Saúde Dr Ricardo Jorge, Porto, Portugal; tt EPIUnit - Instituto de Saude Publica, Universidade do Porto, Porto, Portugal; uu Laboratory for Integrative and Translational Research in Population Health (ITR), Porto, Portugal; vv Faculty of Geography, Babes-Bolay University, Cluj-Napoca, Romania; ww Department of Environmental Health. Rollins School of Public Health, Emory University, Atlanta, Georgia; xx Department of Earth Sciences, University of Torino, Torino, Italy; yy Graduate School of Public Health, Seoul National University, Seoul, South Korea; zz School of Biomedical Convergence Engineering, Pusan National University, Yangsan, South Korea; aaa Department of Statistics and Computational Research, Universitat de València, València, Spain; bbb CIBER of Epidemiology and Public Health, Madrid, Spain; ccc Department of Public Health and Clinical Medicine, Umeå University, Umeå, Sweden; ddd Institute of Social and Preventive Medicine, University of Bern, Bern, Switzerland; eee Oeschger Center for Climate Change Research, University of Bern, Bern, Switzerland; fff University of Basel, Basel, Switzerland; ggg Swiss Tropical and Public Health Institute, Allschwil, Switzerland; hhh Environmental and Occupational Medicine, National Taiwan University (NTU) College of Medicine and NTU Hospital, Taipei, Taiwan; iii National Institute of Environmental Health Science, National Health Research Institutes, Zhunan, Taiwan; jjj Institute of Environmental and Occupational Health Sciences, National Taiwan University College of Public Health, Taipei, Taiwan; kkk Department of Statistics, Informatics, Applications, University of Florence, Florence, Italy; lll Environment & Health Modelling (EHM) Lab, Department of Public Health Environments and Society, London School of Hygiene & Tropical Medicine, London, UK

**Keywords:** Minimum mortality temperature, Climate change, Human adaptation, Temporal change, Heterogeneity

## Abstract

**Background:**

The minimum mortality temperature (MMT) or MMT percentile (MMTP) is an indicator of population susceptibility to nonoptimum temperatures. MMT and MMTP change over time; however, the changing directions show region-wide heterogeneity. We examined the heterogeneity of temporal changes in MMT and MMTP across multiple communities and in multiple countries.

**Methods:**

Daily time-series data for mortality and ambient mean temperature for 699 communities in 34 countries spanning 1986–2015 were analyzed using a two-stage meta-analysis. First, a quasi-Poisson regression was employed to estimate MMT and MMTP for each community during the designated subperiods. Second, we pooled the community-specific temporally varying estimates using mixed-effects meta-regressions to examine temporal changes in MMT and MMTP in the entire study population, as well as by climate zone, geographical region, and country.

**Results:**

Temporal increases in MMT and MMTP from 19.5 °C (17.9, 21.1) to 20.3 °C (18.5, 22.0) and from the 74.5 (68.3, 80.6) to 75.0 (71.0, 78.9) percentiles in the entire population were found, respectively. Temporal change was significantly heterogeneous across geographical regions (*P* < 0.001). Temporal increases in MMT were observed in East Asia (linear slope [LS] = 0.91, *P* = 0.02) and South-East Asia (LS = 0.62, *P* = 0.05), whereas a temporal decrease in MMT was observed in South Europe (LS = −0.46, *P* = 0.05). MMTP decreased temporally in North Europe (LS = −3.45, *P* = 0.02) and South Europe (LS = −2.86, *P* = 0.05).

**Conclusions:**

The temporal change in MMT or MMTP was largely heterogeneous. Population susceptibility in terms of optimum temperature may have changed under a warming climate, albeit with large region-dependent variations.

**What this study adds:**

This study examines the geographical heterogeneity in the temporal changes in minimum mortality temperature (MMT) across 34 countries (699 communities) over the 30-year period spanning 1986–2015. This study attempts to investigate both the pooled evidence and the heterogeneity in the temporal change in MMT on a global scale. Our findings suggest an overall increase in MMT globally over the 30 years, with certain country- or region-specific trends displaying both upward and downward movements without discernible patterns. Moreover, the results indicate that the temporal change in MMT is not fully explained by the warming temperatures over time.

## Introduction

A U- or J-shaped association has been established between ambient temperature and human mortality, with a threshold temperature at which the lowest mortality occurs.^1,2^ This threshold value has been referred to as the “minimum mortality temperature” (MMT) and is considered one of the key indicators of population susceptibility to nonoptimum temperatures.^2–4^ MMT has changed over the last few decades,^5–14^ possibly indicating changes in human susceptibility to nonoptimum temperature.

These observed temporal changes are, however, inconsistent across studies. While certain studies have reported increasing MMT, although at different rates,^7–11^ other studies have shown approximately identical or decreasing MMTs over time.^11–13^ Therefore, synthesizing and comparing the results of previous studies are difficult because the study population, study period, causes of death, and analytical methods have varied. To gain a better understanding of the changing MMT, we require a large-scale, multicountry, multicommunity study that involves the analysis of time-series data from multiple populations using a unified statistical analysis framework.

MMT shows a close linear relationship to the average temperature (AT).^1,3,15^ Therefore, MMT can be assumed to increase over time as AT increases under a warming climate. Future changes in MMT have been extrapolated, assuming that the linear relationship between MMT and AT remains unchanged.^15^ However, this relationship has been derived based on the spatial variation in MMT and AT and not on the temporal variation. Further investigations are required to determine the potential relationship between MMT and AT based on temporal variation and/or whether the temporal change in MMT can be fully explained by the temporal change in AT. Other temporally varying factors may affect MMT (e.g., factors that change the temperature–mortality association itself). In such cases, the temporal change in MMT may not strictly reflect the temporal change in AT.

In this study, we investigate the temporal change in MMT and MMT percentile (MMTP) across 699 communities spanning 34 countries by analyzing historical time-series data pertaining to mortality and temperature spanning 1986–2015. The present study aimed to examine (1) how MMT and MMTP have changed over the past decades in the entire study population, (2) heterogeneity in the temporal change in MMT and MMTP across different climate zones, regions, countries, and communities, and (3) whether the temporal change in MMT and MMTP is explained by the temporal change in AT under a warming climate.

## Methods

### Data collection

Data collection was performed as described in previous studies using the Multi-Country Multi-City Collaborative Research Network dataset.^1–3^ In this study, we used daily time-series data for mortality and ambient temperature collected from 699 communities across 34 countries (Table S1 and Figure S1; http://links.lww.com/EE/A298). Data consisted of daily death counts for all causes or nonexternal causes (International Classification of Diseases 9th Revision [ICD-9] 0–799 and 10th Revision [ICD-10] A00–R99) and daily mean temperature for each community. The data collection period varied by country, ranging from 10 (in Moldova, Greece, and Thailand) to 50 (in Norway) years (Figure S2; http://links.lww.com/EE/A298). We restricted our analysis to a 30-year study period spanning 1986–2015; wherever possible, we ensured that the study periods across countries overlapped.

We also collected data on community-specific indicators for climate zones, classifying each community into one of the four Köppen climate zones: tropical, dry, temperate, and continental (Figure S1B; http://links.lww.com/EE/A298). In addition, we created an indicator for geographical regions, classifying each community into one of the 11 regions: North America, Central America, South America, North Europe, Central Europe, South Europe, South Africa, the Middle East, East Asia, South-East Asia, and Australia (Table S1 and Figure S1C; http://links.lww.com/EE/A298).

### Statistical analysis

A two-stage meta-analysis was conducted. In the first stage, we estimated the MMT or MMTP for each community for each of the 5-year nonoverlapping subperiods. In the second stage, community-specific temporally varying MMTs or MMTPs were pooled for the entire study population, as well as by climate zone, geographic region, and country. For computations, we used R statistical software (version 4.0.3; R Development Core Team, Vienna, Austria) with functions from the packages dlnm and mixmeta.

#### Estimating community-specific temporally varying minimum mortality temperature/minimum mortality temperature percentile

We divided the entire study period spanning 1986–2015 into 5-year nonoverlapping subperiods (i.e., 1986–1990, 1991–1995, 1996–2000, 2001–2005, 2006–2010, and 2011–2015). We also conducted a sensitivity analysis using the data from the years 2001–2015 to examine how sensitive the analysis results are to the length of the data period. For each community and subperiod, we fitted a quasi-Poisson regression with splines, enabling overdispersion to estimate the temperature–mortality association as follows. Let *y*_*t*_ be the daily death count on day *t* and **x**_*t*_ = (*x*_*t*_, *x*_*t*−1_, …, *x*_*t*−__𝐿_)^′^ be the vector of daily mean temperatures on day *t* and over the previous 𝐿 days. We used the following generalized linear model with a quasi-Poisson family.


$$\begin{array}{l} y_{t}∼quasi\text{-}Poisson\left(\lambda_{t}\right), \end{array}$$

$$\begin{array}{l} \log\left(\lambda_{t}\right)=\alpha_{0}+s\left(x_{t};\beta \right)+\underset{j=1}{\overset{J}{\sum }}\;h_{j}\left(u_{jt};\gamma_{j}\right),\;\;for\;\;t=L+1,\ldots ,N, \end{array}$$ (1)

where λ_*t*_ = E(𝑦_*t*_) is the expected mortality count on day *t*, 𝛼_0_ is a model intercept, *u*_*jt*_ is the *j*th control variable on day *t*, and $h_{j}\left(\cdot \right)$ is a flexible function to represent the effect of the *j*th control variable, characterized by ***γ***_*j*_. In our analysis, $h_{j}\left(\cdot \right)$ corresponds to a natural cubic B-spline of time with eight degrees of freedom (df) per year to account for seasonality and long-term trends. In addition, we included indicator variables to mitigate the effect of the day of the week.

In Equation (1), s(⋅) is a flexible function to describe a nonlinear and delayed association between temperature and mortality, and we used a distributed lag nonlinear model (DLNM)^16^ as follows. The DLNM defines a cross-basis for temperature and lag: Let $f_{1}\left(\cdot \right),\cdots ,f_{v_{x}}\left(\cdot \right)$ be the basis to describe the nonlinear temperature–mortality association with dimension 𝑣*x*, and let $g_{1}\left(\cdot \right),\cdots ,g_{v_{l}}\left(\cdot \right)$ be the basis to describe the relationship across the lag space. Then, the DLNM for s(⋅) is expressed as follows.

$$\begin{array}{l} s\left(x_{t};\beta \right)=\underset{j=1}{\overset{v_{x}}{\sum }}\;\underset{k=1}{\overset{v_{l}}{\sum }}\;r_{tj}^{\prime }c_{k}\beta_{jk} \end{array}$$ (2)

where $r_{tj}={\left(f_{j}\left(x_{t}\right),\cdot \cdot \cdot ,f_{j}\left(x_{t-L}\right)\right)}^{\prime }$ is the transformed vector using the jth basis 𝑓_*j*_ in the temperature dimension, and $c_{k}={\left(g_{k}\left(0\right),\cdot \cdot \cdot ,g_{k}\left(L\right)\right)}^{\;\prime \;}$ is the transformed vector using the kth basis 𝑔_𝑘_ in the lag dimension. The coefficient vector $\beta ={\left(\beta_{11},\beta_{12,}\ldots ,\beta_{v_{x}v_{l}}\right)}^{\;\prime \;}$ has the length of $v_{x}\times v_{l}$. In our analysis, we used a natural cubic B-spline for temperature with three internal knots placed at the 10th, 75th, and 90th percentiles of location-specific temperature distributions and a natural cubic spline for the lag with an intercept and three internal knots placed at equally spaced values in the log scale. Therefore, 𝑣_*x*_ = 4, 𝑣_𝑙_ = 5, and there are 4 × 5=20 coefficients in s(⋅). The lag was extended to L=21 days to capture long-delayed effects. These specifications were based on the results of model selection in a previous study.^1^

After fitting the quasi-Poisson regression, the coefficients for the cross-basis term were extracted and reduced to summarize the cumulative association between temperature and mortality over the lags^17^ as follows.


$$\begin{array}{l} \theta =M_{1}\beta \end{array}$$

$$\begin{array}{l} V\left(\theta \right)=M_{1}V\left(\beta \right)M_{1}^{\;\prime \;} \end{array}$$ (3)

where ***β*** is the set of 20 coefficients, $M_{1}=I_{v_{x}}⊗1_{L+1}^{\;\prime \;}C$ is a reducing matrix, $\theta ={\left(\theta_{1},\theta_{2}\ldots ,\theta_{v_{x}}\right)}^{\;\prime \;}$ is the set of reduced coefficient with a length of 𝑣_*x*_, and 𝑉(***θ***) is the associated error covariance matrix. The 𝑣_*x*_ = 4 coefficients represent the temperature–mortality association cumulated over the lags.

Using the reduced coefficients and the corresponding standard error matrices, we estimated the MMT and its standard error using a Monte Carlo simulation method.^18,19^ Let $\hat{\theta }$ be an estimate for ***θ*** and $V\left(\hat{\theta }\right)\;$ be the corresponding error matrix. Using $\hat{\theta }$, we obtained a point estimate for the MMT as $\hat{MMT}=argmin_{x}\left(\overset{\nu_{x}}{\underset{k=1}{\sum }}{\hat{\theta }}_{k}f_{k}\left(x\right)\right)$, where 𝑓_𝑘_(*x*) indicates the 𝑘th basis in the temperature dimension evaluated at temperature *x*. Then, using $\hat{\theta }$ and $V\left(\hat{\theta }\right)$, we obtained its standard error through a Monte Carlo sampling method. We first simulated ***θ***^′^𝑠 from a multivariate normal distribution with the mean specified as a pooled estimate $\hat{\theta }$ and the covariance as $V\left(\hat{\theta }\right)$. Then, we estimated the MMT for each simulated ***θ*** as follows:


$$\begin{array}{l} \theta_{\left(\;j\right)}∼N\left(\hat{\theta },V\left(\hat{\theta }\right)\right) \end{array}$$

$$\begin{array}{l} MMT_{\left(\;j\right)}=argmin_{x}\left(\overset{\nu_{x}}{\underset{k=1}{\sum }}\theta_{k,\left(\;j\right)}f_{k}\left(x\right)\right) \end{array}$$ (4)

where (*j*) indicates the *j*th simulation, *x* indicates the observed temperature range, and 𝑓_𝑘_(*x*) indicates the 𝑘th basis in the temperature dimension evaluated at temperature *x*. We obtained 1000 Monte Carlo samples of the MMT through the simulation procedure and used the sample standard deviation as its standard error. We restricted our search for the MMT within the range encompassing the 25th–99th percentiles of the community-specific temperature distribution such that MMT values were not estimated beyond a range where the corresponding statistical uncertainty tended to increase. We also estimated MMT in the range spanning the 1st to 99th percentiles to test the sensitivity of the results to the range restriction. To obtain the MMTP, we converted the estimated MMT to MMTP based on the empirical distribution of observed temperature for each community and subperiod.

#### Pooling the community-specific temporally varying minimum mortality temperature/minimum mortality temperature percentile

We pooled the community-specific MMTs or MMTPs estimated for nonoverlapping subperiods using mixed-effects meta-regression (MEMR).^20^ For the *i*th country, *j*th community, and *w*th period, let ${\hat{MMT}}_{ijw}$ be the MMT and *sd*_*ijw*_ be the corresponding standard error. Let *time*_*w*_ be an integer-valued variable for the *w*th period (1, 2, …, 6). First, we pooled temporally varying MMT’s or MMTP’s for the entire population by fitting the MEMR, where a temporal variable (considering values from 1 to 6 for each subperiod) was included as a linear term.


$$\begin{array}{l} {\hat{MMT}}_{ijw}=\left(\alpha +a_{ij}+c_{i}\right)+\left(\beta +b_{ij}+d_{i}\right)time_{w}+\epsilon_{ijw} \end{array}$$

$$\begin{array}{l} (a_{ij},b_{ij})\prime ∼N\left(0,{\;\Psi \;}^{\left(2\right)}\right),\;\left(c_{i},d_{i}\right)\prime ∼N\left(0,{\;\Psi \;}^{\left(1\right)}\right),\;\\ \;\epsilon_{ijw}∼N\left(0,{sd}_{ijw}^{2}\right) \end{array}$$ (5)

where *𝛼* and *β* are the population intercept and slope terms (i.e., fixed effects), respectively, *a*_*ij*_ and *c*_*i*_ are the community- and country-specific intercept terms, respectively, and *b*_*ij*_ and *d*_*i*_ are the community- and country-specific slope terms, respectively (i.e., random effects). Ψ^(l)^’s are the random effect covariances for level 𝑙. We reflected the two-level structure of country and community. From the fitted model, we obtained the best linear unbiased predictor for country- and community-specific estimates for MMTs and MMTPs.

Second, we pooled temporally varying MMTs or MMTPs by climate zone by separately fitting the MEMR for each of the four climate zones. We fitted the MEMR model in Equation (5) separately for each of the four climate zones to pool the community-specific temporally varying MMT by climate zone. Third, we pooled temporally varying MMTs or MMTPs by geographical region by fitting the MEMR separately for each of the 11 geographical regions. We fitted the MEMR model in Equation (5) separately for each of the eleven geographical regions to pool the community-specific temporally varying MMT by geographical region.

#### Investigating heterogeneity

We investigated heterogeneity in the temporal trends of MMT and MMTP across communities, countries, geographical regions, and climate zones. First, we tested whether the country- and community-specific random slopes improved the model fit by comparing the MEMRs with and without the corresponding random slopes, because the temporal trend was represented by the coefficient for the temporal variable (i.e., linear slope [LS] for time) in MEMR. In addition, we tested whether the temporal trends of MMT and MMTP were heterogeneous across regions and climate zones by comparing the MEMRs with and without the interaction term between region or climate zone indicators and time. Model comparison was based on the likelihood ratio (LR) test and two model fit statistics, the Akaike information criterion and the Bayesian information criterion.

#### Investigating the possible influence of temporal change in average temperature on temporal changes in minimum mortality temperature or minimum mortality temperature percentile

We investigated whether the temporal changes in MMT or MMTP could be explained by temporal changes in AT. We examined the change in the LS estimate for the time variable and its *P* value by fitting the MEMRs with and without temporally varying AT as an additional meta-predictor. The MEMR model with temporally varying AT is as follows.


$$\begin{array}{l} {\hat{MMT}}_{ijw}=\left(\alpha +a_{ij}+c_{i}\right)+\left(\beta +b_{ij}+d_{i}\right)time_{w}+\gamma AT_{ijw}\;\\ \;+\epsilon_{ijw}\;\;\left(model\;1\right) \end{array}$$

$$\begin{array}{l} {\hat{MMT}}_{ijw}=\left(\alpha +a_{ij}+c_{i}\right)+\left(\beta +b_{ij}+d_{i}\right)time_{w}\;\\ \;+\gamma \left(AT_{ijw}-TAT_{ij}\right)+\epsilon_{ijw}\;\;\;\left(model\;1\right) \end{array}$$

where AT refers to the average temperature in the *w*th period at the *j*th community of the *i*th country, TAT refers to the time-averaged average temperature at the *j*th community of the *i*th country. The models for the random terms remain unchanged.

## Results

**Figure 1.**
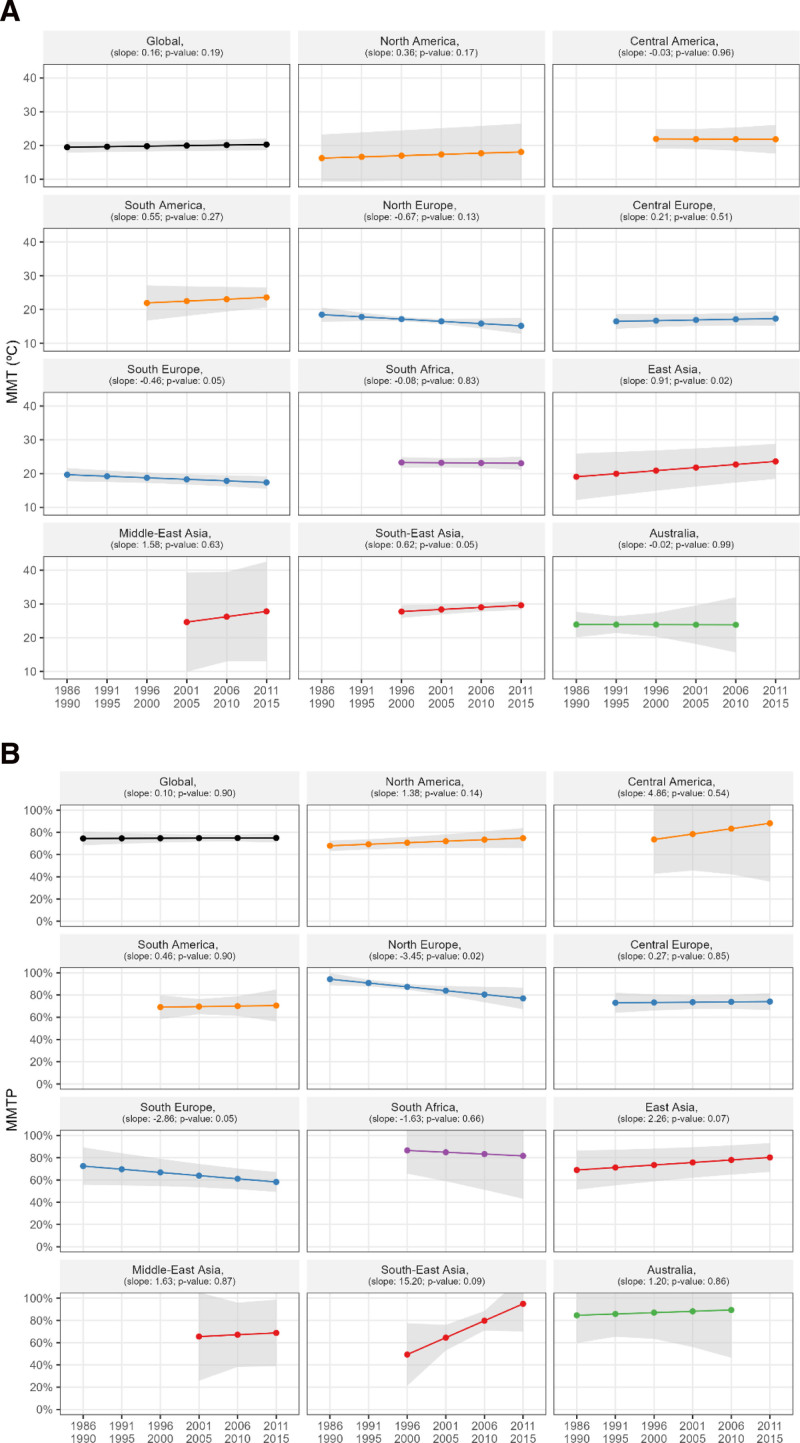
Temporal change in (A) MMT and (B) MMTP in the entire study population (top left) and each of the 11 geographical regions. The linear slope (LS) is presented with the corresponding *P* value.

The top-left panels in Figure 1A and B show the temporal changes in MMT and MMTP, respectively, which were observed in the entire population. Evidence for a temporal increase in MMT and MMTP is shown from 19.5 °C (95% confidence interval [CI]: 17.9, 21.1) to 20.3 °C (95% CI: 18.5, 22.0) and from the 74.5th (95% CI: 68.3, 80.6) to the 75.0th (95% CI: 71.0, 0.78.9) percentiles from the first to last subperiods, respectively. However, both changes were not significant; the slopes for the linear change in MMT and MMTP were estimated to be 0.16 (*P* = 0.19) and 0.1 (*P* = 0.90), respectively. Figure 1A and B also shows region-specific results. Evidence of a temporal increase or decrease in MMT and MMTP was observed in different regions; however, only a subset of them were significant. The temporal increases in MMT were significant in East Asia and South-East Asia (LS = 0.91, *P* = 0.02 and LS = 0.62, *P* = 0.05), and the temporal decrease in MMT was significant in South Europe (LS = −0.46, *P* = 0.05). In addition, the temporal decreases in MMTP were significant in Northern and Southern Europe (LS = −34.51, *P* = 0.02 and LS = −28.56, *P* = 0.05).

**Figure 2.**
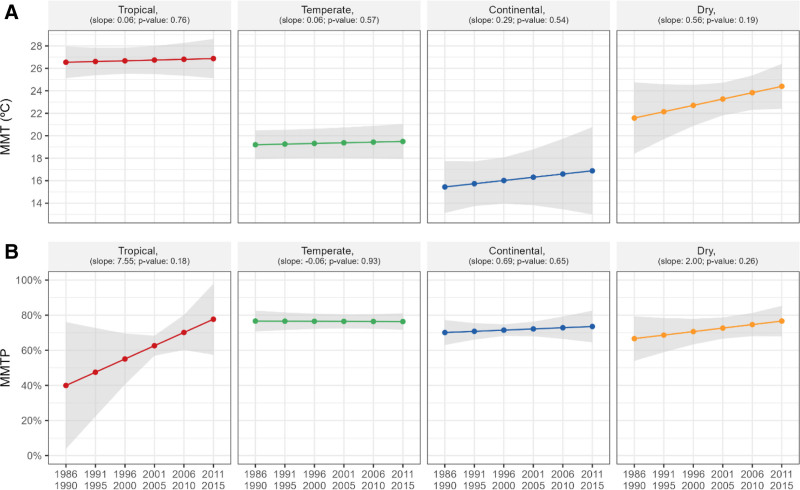
Temporal change in MMT (A) and MMTP (B) in each of the four climate zones. The linear slope (LS) is presented with the corresponding *P* value.

Figure 2 shows the temporal changes in the MMT and MMTP in each of the four climate zones. Temporal increases in MMT were observed in all climate zones; however, none were significant. MMT either increased or remained relatively constant from 26.5 °C to 26.9 °C, 19.2 °C to 19.5 °C, 15.4 °C to 16.9 °C, and 21.6 °C to 24.4 °C in the tropical, temperate, continental, and dry zones, respectively (Figure 2A). The MMTP increased or remained constant from the 39.9th to 77.7th, 76.6th to 76.3th, 70.1th to 73.5th, and 66.6th to 76.6th percentiles (Figure 2B). Figure S3; http://links.lww.com/EE/A298 presents the temporal changes in MMT and MMTP for each of the 34 countries. Overall, country-specific results followed region-specific results; however, the direction and magnitude of temporal changes were heterogeneous across countries in certain geographical regions.

**Figure 3.**
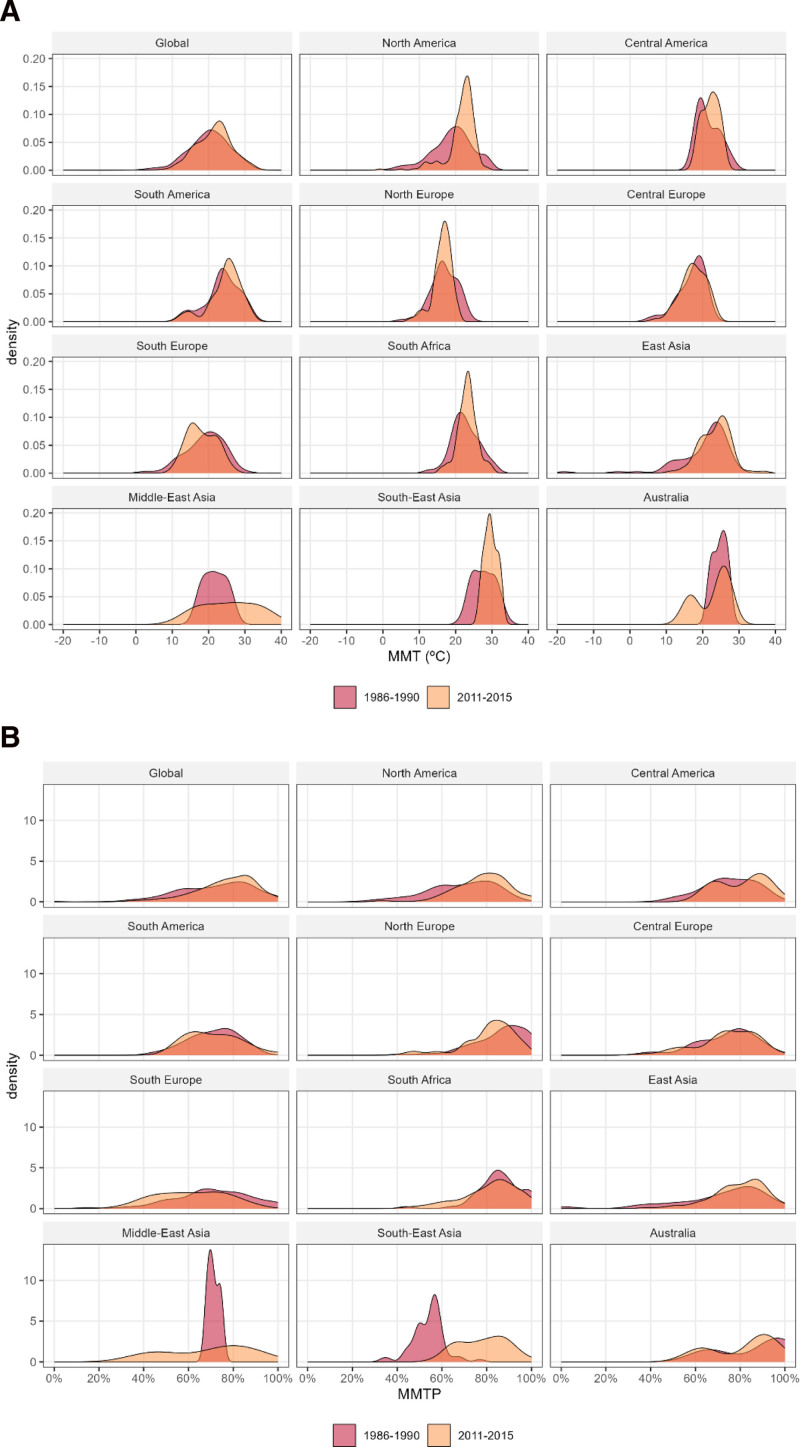
Distribution of the community-specific (A) MMT and (B) MMTP in the first (1986–1990) and last (2011–2015) subperiods in the entire population (top left) and each of the 11 geographical regions.

**Figure 4.**
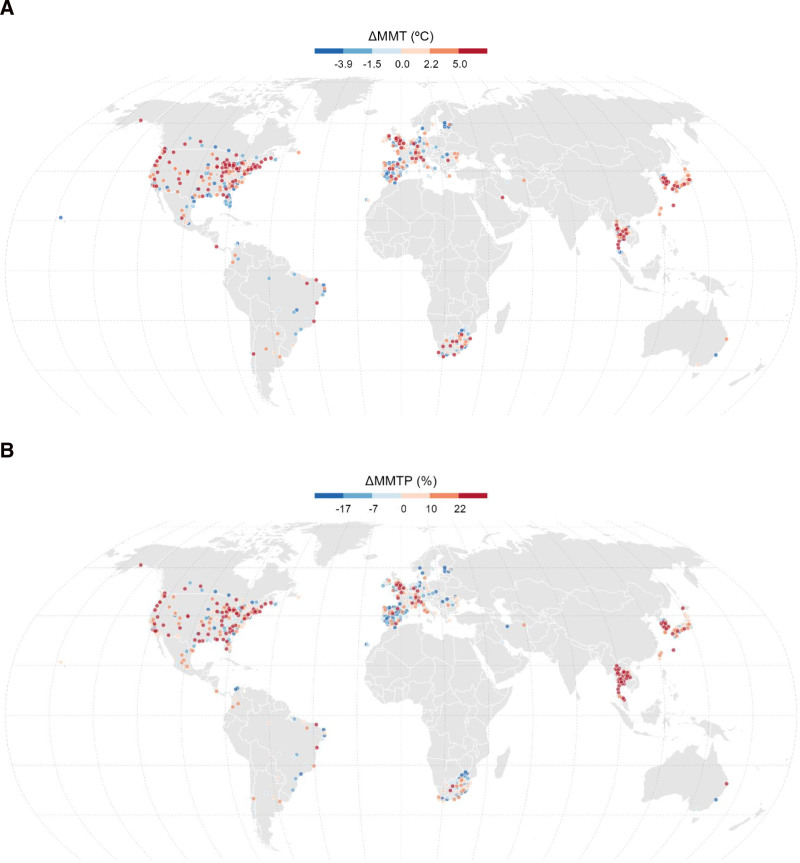
Change in the community-specific (A) MMT and (B) MMTP between the first (1986–1990) and last (2011–2015) subperiods for each of the 677 communities.

Figure 3 displays the distribution of community-specific MMT and MMTP in the first (1986–1990) and last (2011–2015) subperiods for the entire population and for each geographical region. The top-left panels in Figure 3A and B show that in the entire population, the MMT and MMTP distributions moved slightly to the right. The distribution shifted to the right in the regions of North America, Central America, South America, East Asia, and South-East Asia, where evidence for temporal increases in MMT and MMTP was observed (Figure 1). Moreover, the distribution moved to the left in the regions of North and South Europe, wherein evidence for temporal decreases in MMT and MMTP was observed (Figure 1). Figure 4 presents the community-specific changes in MMT and MMTP from the first to last subperiods. Consistent with Figures 1 and 3, increases (red dots) in MMT and MMTP were observed in several communities in North America, Central America, East Asia, and South-East Asia, whereas decreasing values (blue dots) were observed in several communities in North and South Europe.

We tested whether the heterogeneity was statistically significant across climate zones, regions, countries, and communities by comparing the MEMRs with different model specifications using an LR test. The model comparison results are presented in Tables S2 and S3; http://links.lww.com/EE/A298. We found that the heterogeneity was significant across climate zones (*P* < 0.0001 when comparing model 2 to model 1 in Table S2; http://links.lww.com/EE/A298) and regions (*P* < 0.0001 when comparing model 4 to model 3 in Table S2; http://links.lww.com/EE/A298). Next, we confirmed that intercountry heterogeneity was significant by comparing the MEMR with and without country-specific random slopes for time (*P* < 0.0001 when comparing model 0 to model 1 in Table S3; http://links.lww.com/EE/A298). We further confirmed that intercommunity heterogeneity was also significant by comparing the MEMR with and without community-specific random slopes using the LR test (*P* < 0.0001 when comparing model 1 to model 2 in Table S3; http://links.lww.com/EE/A298).

We examined whether the temporal change in MMT or MMTP could be explained, at least in part, by the temporal change in AT by comparing the slope estimates for time (and its *P* value) with and without temporally varying AT in the MEMRs as an additional meta-predictor (Table S4; http://links.lww.com/EE/A298). For MMT, we focused only on East Asia, South-East Asia, and South Europe, where temporal changes were significant. In East Asia, the positive slope estimates decreased slightly with larger *P* values (but were still significant). In South-East Asia, the positive slope increased, but with a larger *P* value, indicating that the temporal increase was not significant. Meanwhile, in Southern Europe, the negative slope further decreased, with a significantly reduced *P* value. For MMTP, we examined Northern and Southern Europe, where the temporal decreases were significant. In Northern Europe, the negative slope slightly increased with an increased *P* value (not significant), while the negative slope in Southern Europe further decreased with a significantly reduced *P* value.

The results of the sensitivity analysis are shown in Figures S4–S7; http://links.lww.com/EE/A298. First, we restricted the MMT to the range spanning the 1st to 99th percentiles of the temperature distribution when estimating the community-specific temporally varying MMT. Figure S4; http://links.lww.com/EE/A298 shows the temporal changes in MMT and MMTP in the entire population and for each geographical region. Figure S5; http://links.lww.com/EE/A298 shows the empirical distribution of MMT and MMTP in the first and last subperiods globally and for each region. The results closely resembled those presented in Figures 1 and 3. Additionally, we limited the study period to the years 2001–2015. Figures S6 and S7; http://links.lww.com/EE/A298, respectively, show the temporal change in MMT and MMTP by region and climate zone. Figure S6; http://links.lww.com/EE/A298 shows that the results were generally similar to Figure 1. Nevertheless, in several regions like North America, North Europe, and Australia, the restricted period analysis has captured short-term fluctuations more than long-term trends with larger statistical uncertainty. Similarly, results in Figure S7; http://links.lww.com/EE/A298 are generally similar to those in Figure 2 except that in the Continental zone, the shorter-period analysis showed a decreasing trend while the longer-period analysis led to an increasing trend.

## Discussion

We conducted an observational study to investigate the temporal changes in MMT and MMTP and their heterogeneity in 699 communities across 34 countries spanning 1986–2015. This is the first global-scale study to synthesize inconsistent findings on temporal changes in MMT and MMTP from multiple populations. Our pooled results showed that MMT and MMTP may have remained at the same level throughout the study period in the entire population. However, the direction and magnitude of the temporal changes in MMT and MMTP were largely heterogeneous across climate zones, geographical regions, countries, and communities. Among the heterogeneous results, significant increases in MMT were observed in East Asia and South-East Asia, and significant decreases in MMT and MMTP were observed in North and South Europe. We further investigated whether the temporal changes in MMT or MMTP could be explained by the temporal changes in AT. The results showed that the changes in AT might not fully explain the region-wise changes in MMT or MMTP.

Although insignificant, MMT might have increased slightly (LS = 0.16, *P* = 0.19), while MMTP might have remained constant (LS = 0.1, *P* = 0.90) globally. Potentially, this can be interpreted as warming temperatures might have increased MMT if MMTP was fixed at a certain percentile and all other factors remained constant.^13^ However, our pooled results should not be interpreted as a single general trend but rather as the result of cancelations of temporal increases and decreases over multiple locations. As discussed in the following paragraphs, heterogeneous evidence of temporal increases and decreases was found depending on the region, country, and community.

We found that the temporal change in MMT or MMTP was heterogeneous across different climate zones. The LSs for temporal change were estimated to be positive, although not significant. However, the slope estimates differed significantly across climate zones, suggesting that the magnitude of temporal increases varied across climate zones.

Regionally, greater heterogeneity was observed. Temporal increases in MMT were found in East and South-East Asia. We propose two plausible interpretations: either populations have become less susceptible to heat or warming climates have increased MMT. To test whether warming climates have raised MMT, we examined whether the temporal increase in MMT can be explained by the temporal increase in AT; the results showed region-wide variation. After adjusting for temporally varying AT, the temporal increase in MMT became nonsignificant in South-East Asia but not in East Asia. This indicates that the temporal increase in MMT is not fully explained by the change in AT in East Asia; the former interpretation of population adaptation might be more plausible. Populations may have adapted to increasing temperatures via multiple mechanisms, such as physiological adaptation, behavioral changes, technological adaptation, and changes in public health infrastructure.^8–11^

Alternatively, temporal decreases in MMT and MMTP were observed in Northern and Southern Europe. These results are consistent with those of recent studies conducted in Spain.^11–13^ A plausible hypothesis is that MMTP can decrease because of warming temperature without changing the MMT value. However, in Southern Europe, both MMT and MMTP decreased, indicating that the temperature–mortality association has changed such that both metrics shifted to the left with a warming climate. Recently, it has been observed that the temperature–mortality association curve flattens at its lower end, thereby changing the association from a V-shape to a U-shape, which resulted in a reduction in MMT with greater levels of uncertainty.^13^

The country-specific results (Figure S3; http://links.lww.com/EE/A298) suggest greater heterogeneity across countries, which is supported by the LR test for including country-specific random slopes in the MEMR model. Some of our country-specific results are consistent with those of previous studies. We observed that MMT and MMTP increased in Japan and France and decreased in Spain; both trends were consistent with the findings of previous studies.^8,9,13^ However, some of our results do not support those of previous studies conducted in the Netherlands,^10^ Sweden,^7^ and Spain.^11^ We found that MMT has remained constant, whereas MMTP has decreased in the Netherlands and Sweden; however, it has been previously reported that MMT increased in those two countries. The study population could be a source of discrepancy; while the earlier study^10^ only considered the elderly population, we included the total population. Moreover, while the study in Sweden^7^ investigated trends from earlier periods (i.e., 1901–2009), we investigated the more recent period spanning 1986–2015. Their results indicated that MMT remained similar at the end of the study period, which might suggest that MMT would remain after that. In addition, while the study from Spain focused on cardiovascular mortality,^11^ we considered total mortality. Similar results have been reported for respiratory and total mortality in other studies from Spain.^12,13^

Recently, a multicountry, multicommunity study was conducted to investigate the spatial heterogeneity of MMT and the underlying predictors that explain spatial heterogeneity.^3^ AT was found to be the strongest predictor of spatial variation in MMT. Moreover, MMT and AT showed a linear relationship with the LSs varying across different climate zones. However, in any of the previous studies, whether the temporal variation of AT explains the temporal variation of MMT remained unexplored. This study is the first attempt to examine this hypothesis, and the results showed that depending on the regions, the change in AT might not fully explain the change in MMT or MMTP. We can interpret this as population susceptibility that might have changed due to other temporally varying factors than the AT, such as physiological adaptation, behavioral changes, technological adaptation, and changes in public health infrastructure.

Various previous studies examined the hypothesis related to potential drivers or mechanisms by which populations adapt to climate change. One study showed that changes in climate, demographic, and socioeconomic factors may be associated with the changes in MMT, suggesting that those factors may be potential drivers of the adaptation of the Japanese population to heat.^8^ Other studies found that there exists an association between AC prevalence and heat risk in Japan and the United States.^21,22^ A multicountry study proposed that the development of public health strategies may have mitigated heat-related climate change impacts.^23^ Another multicountry research on summer heat indicated that several factors, such as true acclimatization, adaptive behaviors, or harvesting effects, could explain the changes in susceptibility to heat.^6^ In this study, we could not investigate the underlying mechanism of climate change adaptations more specifically because relevant data were available only for a limited number of countries. Certainly, this should be one of the follow-up topics to be studied in the near future.

As indicated in previous studies, MMT estimates can be affected by the statistical methods used.^7,10^ Various methodologies can be employed for estimating MMT. The majority of older studies used Poisson regression with a piecewise linear spline, which assumes a V-shaped association between temperature and mortality. Given the prevalence of nonlinear lag effects, modeling nonlinear associations with splines has become more common in recent studies. A widely used method is the DLNM, which assumes a nonlinear and nonlinearly delayed association between temperature and mortality. A recent study compared these methods and evaluated how they affect the results of the temporal shift in MMT in the Netherlands,^10^ concluding that the estimates differ depending on the statistical method used. In the present study, we adopted the DLNM approach, as it is a less restrictive and more flexible method to model the temperature–mortality association. When using the DLNM approach, model selection is crucial because the results can be sensitive to the complexity of the splines (i.e., the type of spline, degrees of freedom, knot locations, and maximum lag). The present study referred to the choices selected in a previous study,^1^ where various specifications were evaluated in modeling multicountry multicommunity data.

In addition to statistical methods, temporally varying MMT estimates can be affected by the definition of the temporal unit to examine the temporal shift.^7^ In several studies, moving subperiods (i.e., overlapping subperiods) were used with different sizes of moving windows.^7,9^ In other studies, nonoverlapping subperiods of varying lengths were used.^23^ Alternatively, the entire data period can be modeled simultaneously using a time-varying DLNM approach.^8^ We used the second approach (i.e., 5-year nonoverlapping subperiods) because independent temporal estimates could be obtained to satisfy the assumption required to execute the MEMR in the second stage. In addition, the length of the temporal unit was carefully selected to stably estimate the temporal trend.

In addition, quantifying the uncertainty of the MMT estimate (i.e., CI) is crucial for two reasons. First, certain studies have provided only point estimates,^11,12^ which can be misleading because the CI can be very wide^18^ if the temperature–mortality association is J- or U-shaped with a wide bottom. Consequently, presenting only point estimates without uncertainty can result in misleading conclusions regarding the temporal shift of MMT. Second, when pooling the community-specific temporally varying MMT estimates through MEMR, the uncertainty should be incorporated into the model. In the present study, we adopted a Monte Carlo simulation approach that had been previously introduced in studies^18,19^ to generate CIs for point estimates of MMT, a method that has become a standard practice in temperature–mortality studies.

This study has several limitations. First, the data collection period varied by country and ranged between 10 and 30 years. This indicates that, when pooling the temporally varying MMT across communities or countries, the available information varied over time. To ensure the reasonability of the pooled results while attempting to maintain a sufficiently extended period conducive to stable temporal shift estimation, we restricted our analysis to the study period spanning 1986–2015; this ensured that, wherever possible, the periods overlap across countries, enabling the analysis of approximately 30 decadal periods of data. Nevertheless, the pooled results should be interpreted with caution because of the imbalanced data over time. Second, community types differ across countries, cities, regions, provinces, and prefectures. In addition, in certain countries, communities represent the entire population of the country, while only selected locations are included in other countries. Therefore, country-specific results should be carefully interpreted, noting that not all communities are included to produce pooled results in certain countries. Third, we assumed a linear trend for temporal changes in MMT or MMTP. Alternatively, we used categorical indicators for the subperiod and examined an unstructured trend from which evidence showing a nonlinear trend was observed (results not shown). Although nonlinear trends can be modeled, we assumed linearity because the study period was relatively short (approximately three decades), and only six subperiods were defined as temporal units. In future studies, when more extended data periods are available, more complex temporal trends are worth investigating. Fourth, we did not investigate the potential role of humidity in the temporal changes of MMT because the data for humidity were available for a limited number of countries. A recent study,^24^ however, showed that the role of humidity in the temperature–mortality association is not significant by analyzing the data for a subset of our study population, which implies that humidity may not be a relevant factor that may explain the temporal change in MMT. Finally, subgroup (i.e., by sex or age group) analysis and cause-specific analysis are warranted to scrutinize the underlying factors for the temporal trend of MMT and MMTP.

## Conclusions

We conducted a global-scale, multicountry observational study to synthesize inconsistent findings on the temporal changes in MMT and MMTP in multiple populations. The results showed that MMT and MMTP may have changed or remained constant globally over the study period, but the direction and magnitude of the temporal changes have been largely heterogeneous across climate zones, geographical regions, countries, and communities. The results suggest that human adaptation, in terms of optimum temperature, might largely depend on climatic conditions and regional and country-specific characteristics.

## Conflicts of interest statement

The authors declare that they have no conflicts of interest with regard to the content of this report.

## Supplementary Material


