# Supplementary material for: Temporal change in minimum mortality temperature under changing climate: A multicountry multicommunity observational study spanning 1986–2015
Source: Environ Epidemiol. 2024 Sep 30;8(5):e334. doi: 10.1097/EE9.0000000000000334 (PMC11567688; doi:10.1097/EE9.0000000000000334)

Supplementary material

Temporal change in minimum mortality temperature under changing climate: A multi-country multi-community observational study spanning 1986–2015

List of contents

Tables

Table S1. Basic information and summary statistics of temperature and mortality for 34 countries

Table S2. Model comparison for investigating heterogeneity in the temporal change in MMT across climate zones and geographical regions

Table S3. Model comparison for investigating heterogeneity in the temporal change in MMT across countries and communities

Table S4. Model comparison for examining whether the temporal change in (A) MMT and (B) MMTP is explained by the temporal change in average temperature (AT)

Figures

Figure S1. Locations of the 699 communities in a world map with the (A) average temperatures, (B) climate zones, and (C) geographical regions

Figure S2. Data collection period for each of the 34 countries. The vertical lines indicate the analysis periods

Figure S3. Temporal change in (A) MMT and (B) MMTP for each of the 34 countries

Figure S4. Temporal change in (A) MMT and (B) MMTP in the entire study population (top-left) and each of the 11 geographical regions from the sensitivity analysis using different range restriction (1st to 99th) for MMT

Figure S5. Distribution of the community-specific (A) MMT and (B) MMTP in the first (1986–1990) and last (2011–2015) subperiods in the entire population (top-left) and each of the 11 geographical regions from the sensitivity analysis using different range restriction (1st to 99th) for MMT

Figure S6. Temporal change in (A) MMT and (B) MMTP in the entire population (top-left) and each of the 11 geographical regions from the sensitivity analysis using a different study period from 2001 to 2015

Figure S7. Temporal change in MMT (top) and MMTP (bottom) in each of the four climate zones from the sensitivity analysis using a different study period from 2001 to 2015

Table S1. Basic information and summary statistics of temperature and mortality for 34 countries

Country	Region	Latitude[a] (°)	Longitude[a] (°)	Number of communities by climate zone[b]	Number of communities	Study period (years)	Total number of deaths	Average number of daily deaths	Average temperature (□)
Finland	North Europe	60.17	24.94	D: 1	1	1994–2014 (21 y)	153308	19.99	6.22
Norway	North Europe	59.91	10.75	D: 1	1	1969–2018 (50 y)	271378	14.86	4.78
Estonia	North Europe	58.99	26.32	D: 5	5	1997–2018 (22 y)	167752	20.88	6.16
Sweden	North Europe	57.54	14.35	C: 2, D: 1	3	1990–2016 (27 y)	717294	72.73	8.54
Ireland	North Europe	53.62	−7.34	C: 6	6	1984–2007 (24 y)	1058215	120.72	9.74
United Kingdom (UK)	North Europe	52.38	−1.28	C: 70	70	1990–2016 (27 y)	6167130	625.34	10.46
Netherland	North Europe	52.15	5.71	C: 4	5	1995–2016 (22 y)	3050053	379.55	10.32
Germany	Central Europe	51.22	9.89	C: 12	12	1993–2015 (23 y)	3105865	369.75	10.25
Czech Republic	Central Europe	49.51	15.94	C: 4	4	1994–2015 (22 y)	711910	88.6	9.09
France	Central Europe	46.91	3.13	C: 18	18	2000–2014 (15 y)	1639262	299.19	12.64
Switzerland	Central Europe	46.89	7.87	C: 7, D: 1	8	1995–2013 (19 y)	243638	35.11	10.45
Moldova	Central Europe	46.72	28.4	C: 3, D: 1	4	2001–2010 (10 y)	59906	16.4	10.69
Canada	North America	46.42	−88.13	C: 3, D: 23	26	1986–2015 (30 y)	3733749	340.76	6.84
Romania	Central Europe	45.46	25.57	C: 5, D: 3	8	1994–2016 (23 y)	951146	113.22	10.83
Italy	South Europe	42.56	12	C: 12	12	1987–2010 (24 y)	820390	93.59	15.06
Spain	South Europe	39.92	−3.87	B: 7, C: 45	52	1990–2014 (25 y)	3017110	330.42	15.51
Portugal	South Europe	39.58	−8.31	C: 5	5	1980–2018 (39 y)	1750670	122.9	15.76

United States (US)	North America	38.09	−90.54	A: 4, B: 17, C: 127, D: 63	211	1973–2006 (34 y)	38040638	3063.35	13.96
Greece	South Europe	37.98	23.72	C: 1	1	2001–2010 (10 y)	287969	78.85	18.67
South Korea	East Asia	36.21	127.9	C: 19, D: 17	36	1997–2018 (22 y)	3070357	382.12	13.18
Iran	Middle-East Asia	35.99	55.51	B: 1, C: 1	2	2002–2015 (14 y)	817913	159.97	16.88
Chile	South America	−35.46	−71.74	C: 4	4	2004–2014 (11 y)	325462	81	13.37
Japan	East Asia	35.38	136.04	C: 41, D: 6	47	1972–2015 (44 y)	39917611	2483.83	15.12
Australia	Australia	−33.04	149.74	C: 3	3	1988–2009 (22 y)	1177950	146.58	18.11
Argentina	South America	−32.99	−61.07	C: 3	3	2005–2015 (11 y)	686333	170.86	18.16
Kuwait	Middle-East Asia	29.38	47.99	B: 1	1	2000–2016 (17 y)	73748	11.88	27.11
South Africa	South Africa	−28.77	27.03	A: 1, B: 23, C: 28	52	1997–2013 (17 y)	8509130	1370.45	17.96
Paraguay	South America	−25.3	−57.64	C: 1	1	2004–2019 (16 y)	48037	8.22	23.31
Taiwan	South-East Asia	23.94	120.82	A: 1, C:2	3	1994–2014 (21 y)	1209573	157.7	23.97
Mexico	Central America	23.6	−102.89	B: 5, C: 5	10	1998–2014 (17 y)	2980086	479.96	18.36
Thailand	South-East Asia	14.52	101.23	A: 62	62	1999–2008 (10 y)	1827853	500.37	27.59
Brazil	South America	−12.59	−44.34	A: 14, C: 4	18	1997–2011 (15 y)	3401136	620.87	24.58
Costa Rica	South America	9.63	−84.25	A: 1	1	2000–2017 (18 y)	31117	4.73	22.68
Colombia	South America	7.13	−75.31	A: 4, C: 1	5	1998–2013 (16 y)	956539	163.68	23.41

[a] Longitude and latitude of the capital city of each country. [b]A: Tropical, B: Dry, C: Temperate, D: Continental.

Table S2. Model comparison for investigating heterogeneity in the temporal change in MMT across climate zones and geographical regions

MEMR for Global	Specification	df	I^2 (%)	AIC	BIC	p-value from LR test
Model 0*	Time	8	85.36302	19010.85	19058.28	
Model 1	Model 0 + Climate zone	11	80.88020	18925.48	18990.69	<0.0001
Model 2	Model 1 + Time*Climate zone	14	80.80552	18909.43	18992.41	<0.0001
Model 3	Model 0 + Region	18	76.57001	18914.29	19020.95	<0.0001
Model 4	Model 3 + Time*Region	28	75.10999	18868.53	19034.34	<0.0001

* Model 0 corresponds to the MEMR of formula (5) in the Supplementary Materials.

Table S3. Model comparison for investigating heterogeneity in the temporal change in MMT across countries and communities

MEMR for Global	Specification	df	I^2 (%)	AIC	BIC	p-value from LR test
Model 0*	Fixed effects + random intercepts only	4	85.3630	19356.54	19380.25	
Model 1	Model 0 + country-specific random slope	6	85.3630	19297.17	19326.81	<0.0001
Model 2**	Model 1 + community-specific random slope	8	85.3630	19010.85	19058.28	<0.0001

* Model 0 corresponds to the MEMR of formula (5) with no random slopes in the Supplementary Materials.

** Model 2 corresponds to the MEMR of formula (5) in the Supplementary Materials.

Table S4. Model comparison for examining whether the temporal change in (A) MMT and (B) MMTP is explained by the temporal change in average temperature (AT)

(A)MMT

Region	Specification	Coefficient for Time	p-value	AIC
South Europe	Model 0*: time variable as the only fixed effect	-0.460	0.049	2124.548
	Model 1: Model 0 + the average temperature as a fixed effect	-0.628	0.016	2073.729
	Model 2: Model 0 + (average temperature – total average temperature) as a fixed effect	-0.863	0.003	2115.185
East Asia	Model 0: time variable as the only fixed effect	0.912	0.019	3205.493
	Model 1: Model 0 + the average temperature as a fixed effect	0.844	0.034	3190.877
	Model 2: Model 0 + (average temperature – total average temperature) as a fixed effect	0.895	0.028	3202.404
South-East Asia	Model 0: time variable as the only fixed effect	0.622	0.052	535.2028
	Model 1: Model 0 + the average temperature as a fixed effect	0.681	0.030	532.0968
	Model 2: Model 0 + (average temperature – total average temperature) as a fixed effect	0.756	0.162	533.7393

* Model 0 corresponds to formula (5) in the Supplementary Materials.

(B)MMTP

Region	Specification	Coefficient for Time	p-value	AIC
North Europe	Model 0*: time variable as the only fixed effect	-0.035	0.019	17.98343

	Model 1: Model 0 + the average temperature as a fixed effect	-0.034	0.026	21.09305
	Model 2: Model 0 + (average temperature – total average temperature) as a fixed effect	-0.023	0.100	17.83158
South Europe	Model 0: time variable as the only fixed effect	-0.029	0.054	183.9949
	Model 1: Model 0 + the average temperature as a fixed effect	-0.032	0.030	176.8756
	Model 2: Model 0 + (average temperature – total average temperature) as a fixed effect	-0.046	0.007	183.1734

* Model 0 corresponds to formula (5) in the Supplementary Materials.

Figure S1. Locations of the 699 communities in a world map with (A) average temperature, (B) climate zones, and (C) geographic regions

(A)

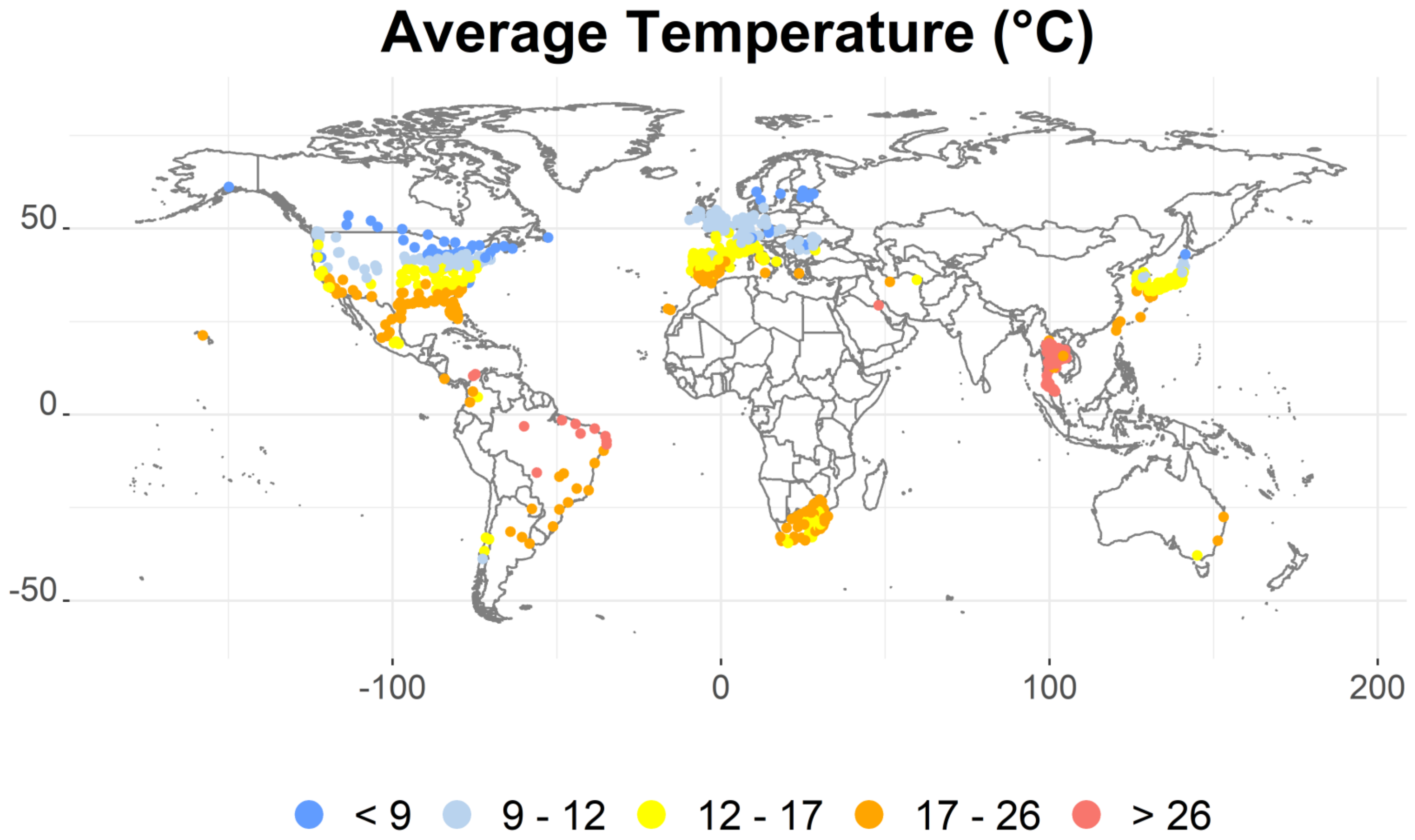

(B)

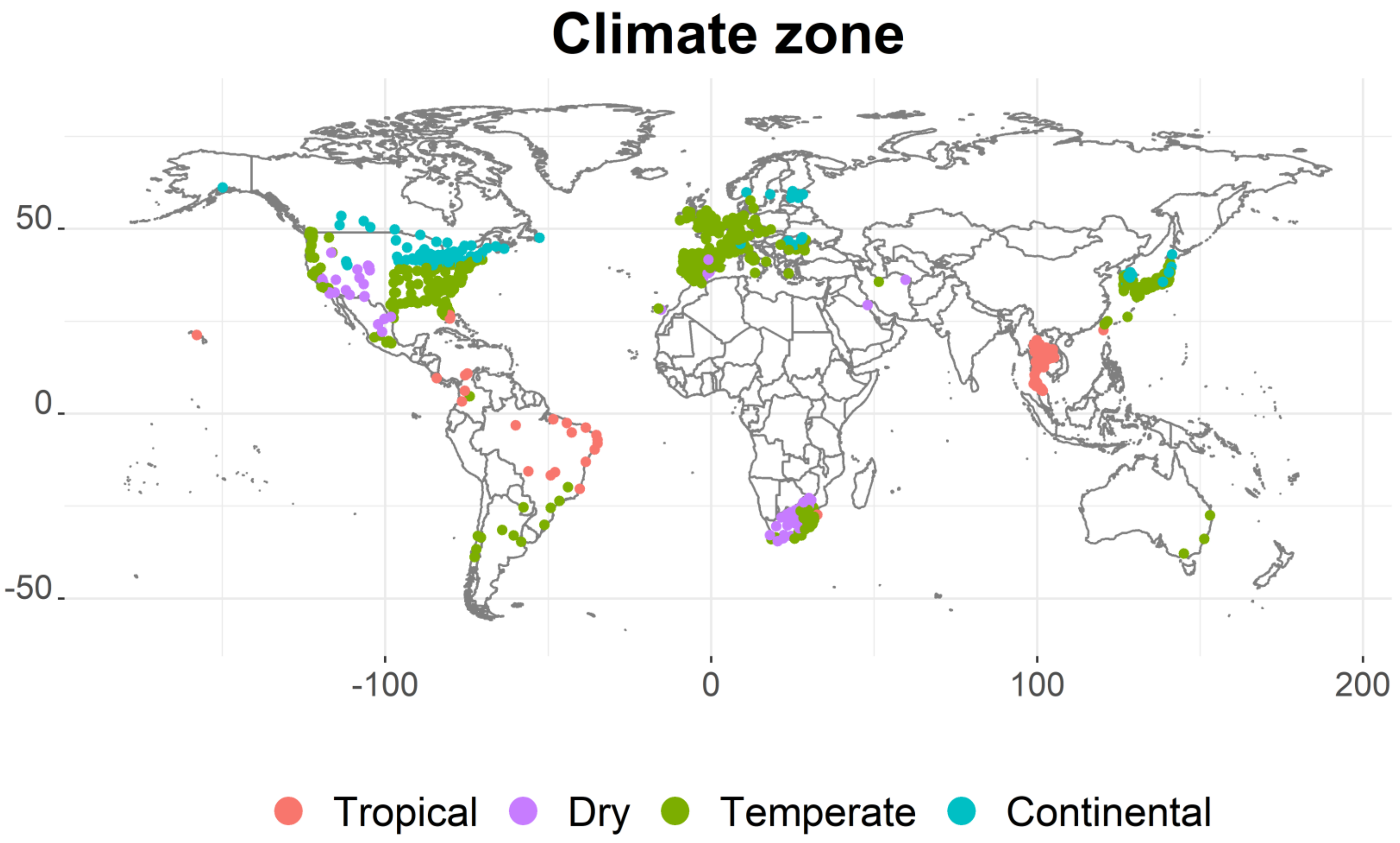

Climate zone
50
0
-50
-100
0
100
200
Tropical
Dry
Temperate
Continental

(C)

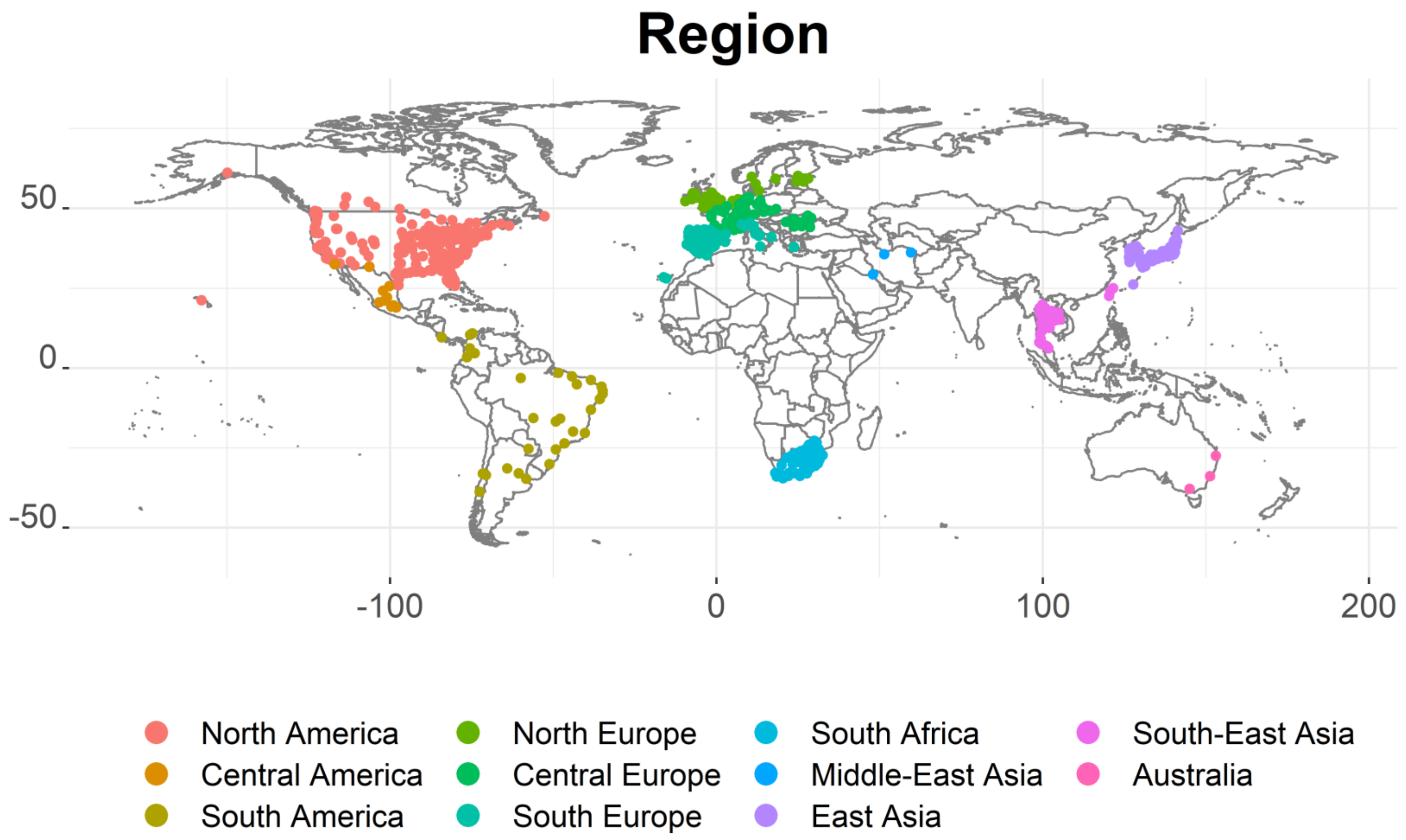
Region
50
0
-50
-100
0
100
200
North America
Central America
South America
North Europe
Central Europe
South Europe
South Africa
Middle-East Asia
East Asia
South-East Asia
Australia

Figure S2. Data collection period for each of the 34 countries. The vertical lines indicate the analysis period

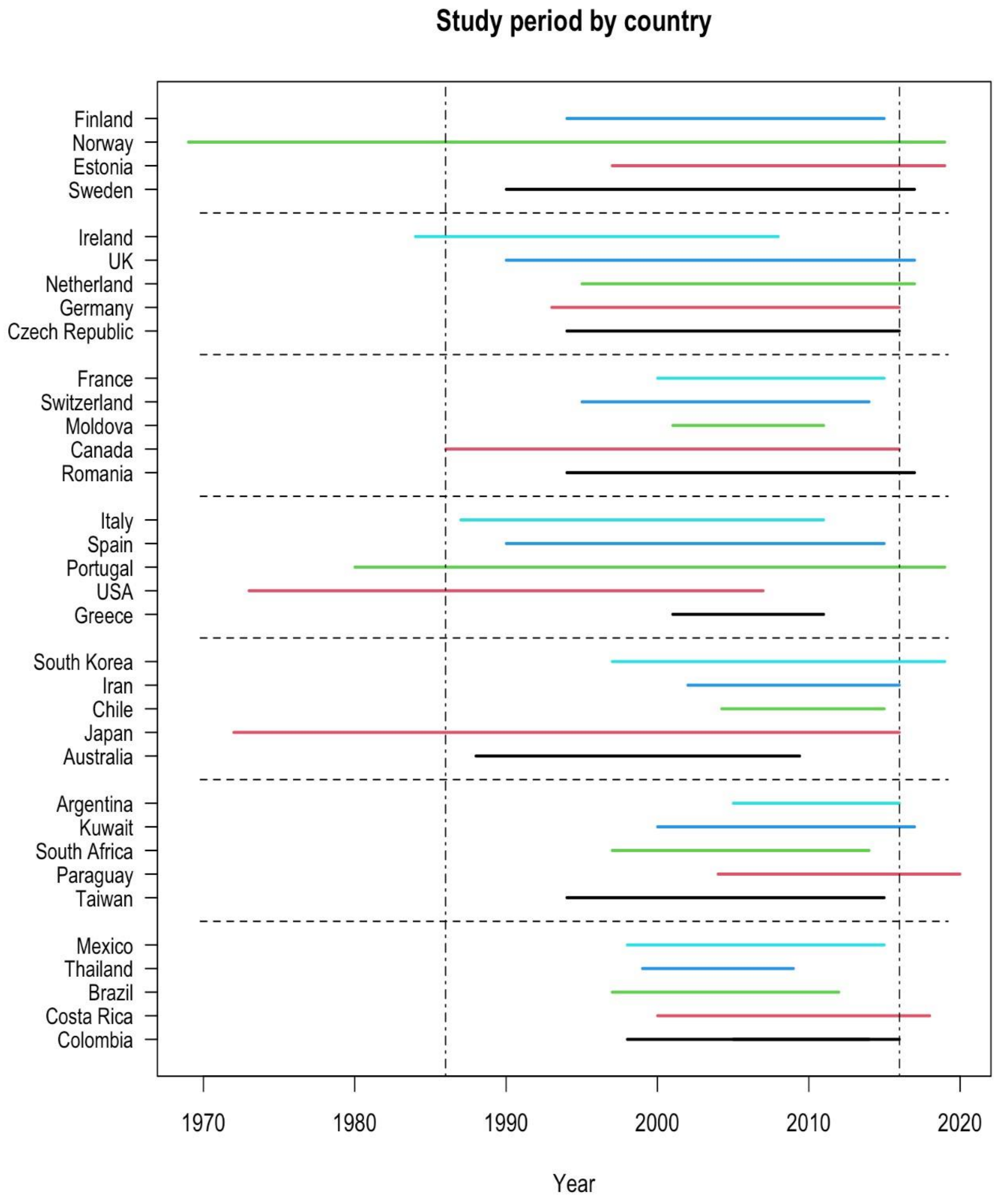

Figure S3. Temporal change in (A) MMT and (B) MMTP for each of the 34 countries

(A)

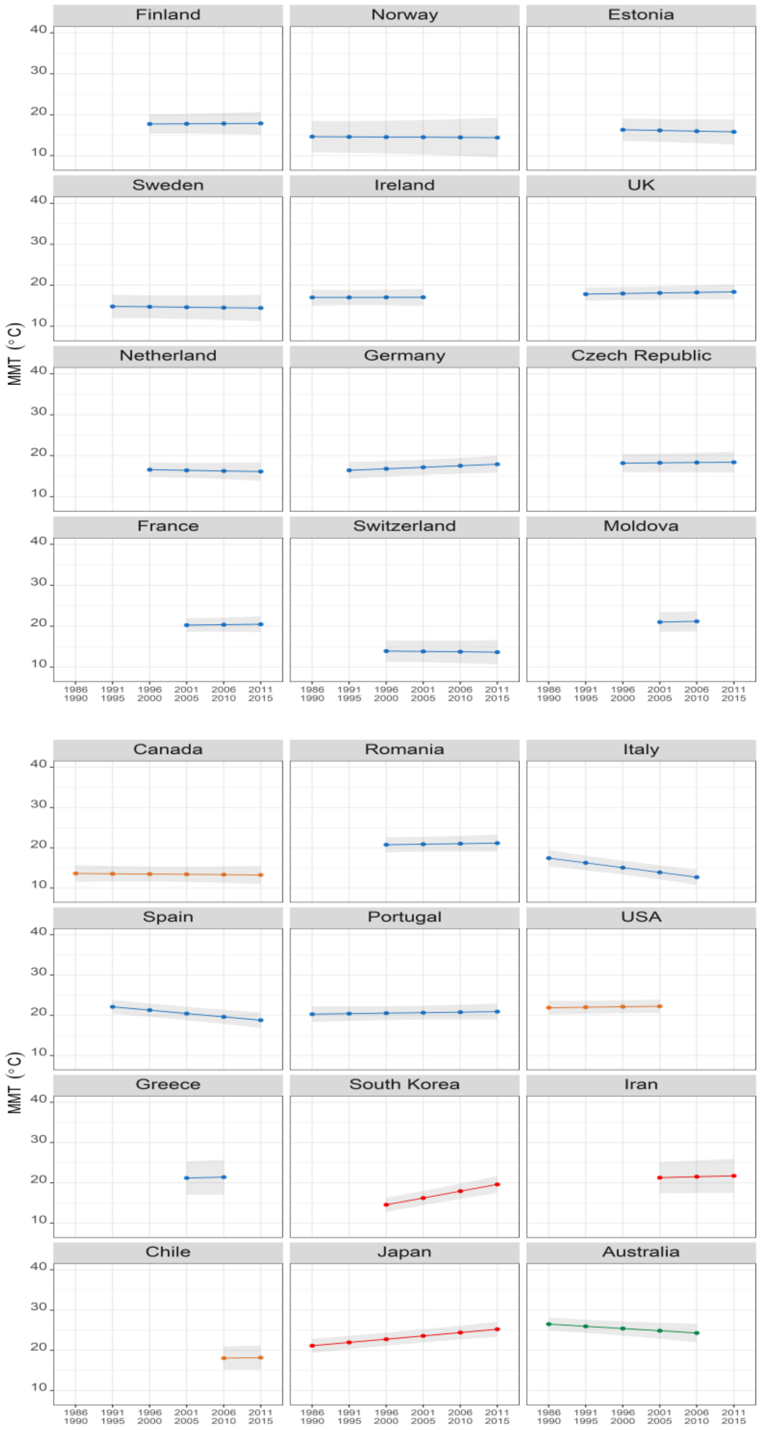

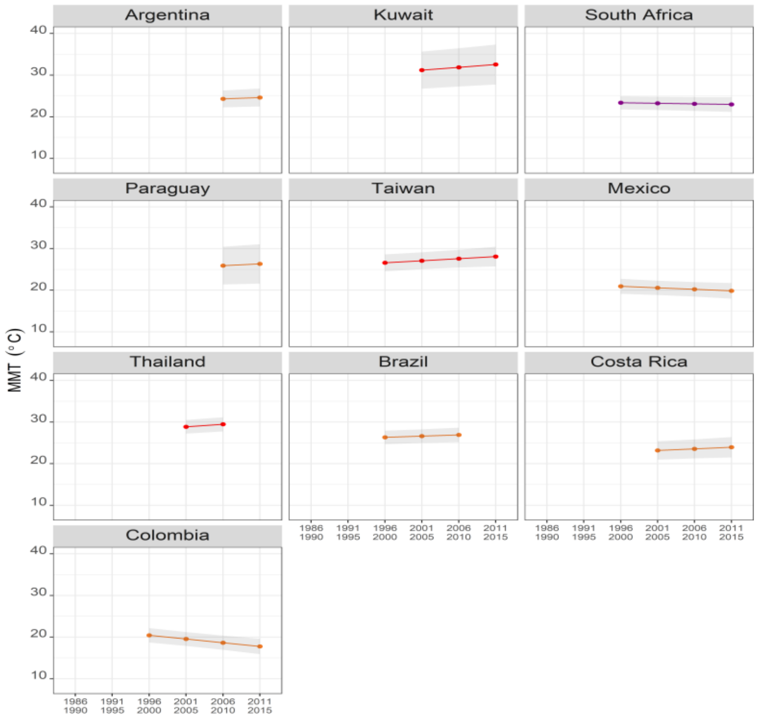
Argentina
Kuwait
South Africa
Paraguay
Taiwan
Mexico
Thailand
Brazil
Costa Rica
Colombia
MMT (°C)
40
30
20
10
1986 1990
1991 1995
1996 2000
2001 2005
2006 2010
2011 2015

(B)

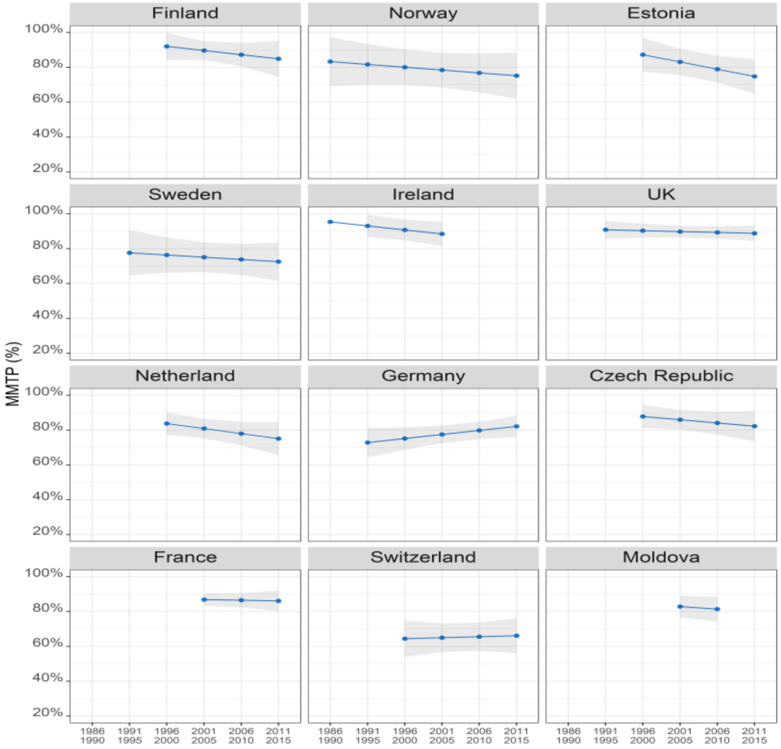
Finland
Norway
Estonia
Sweden
Ireland
UK
Netherland
Germany
Czech Republic
France
Switzerland
Moldova
MMTP (%)
100%
80%
60%
40%
20%
1986 1990
1991 1995
1996 2000
2001 2005
2006 2010
2011 2015

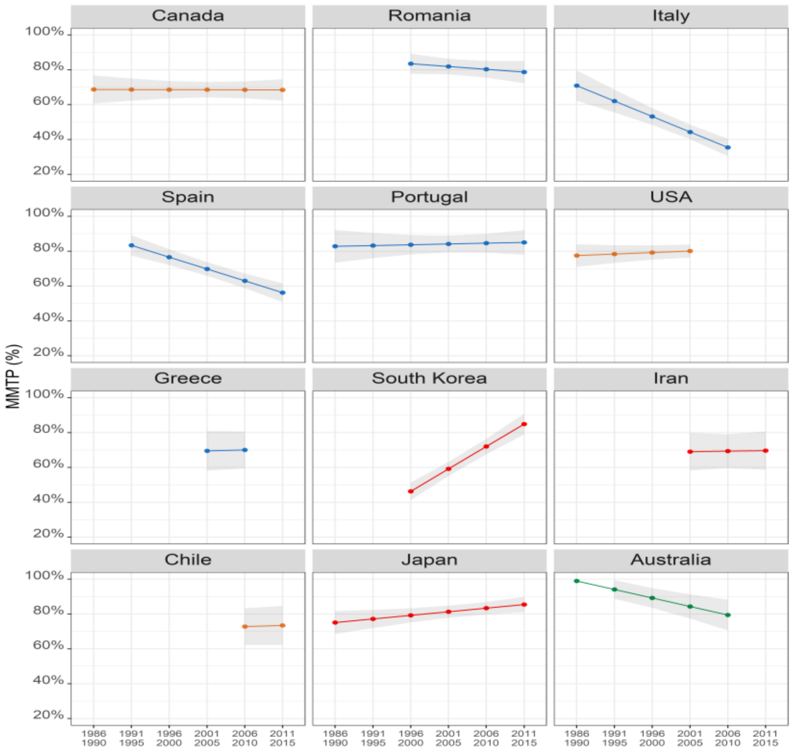
Canada
Romania
Italy
Spain
Portugal
USA
Greece
South Korea
Iran
Chile
Japan
Australia
MMTP (%)
100%
80%
60%
40%
20%
1986 1990
1991 1995
1996 2000
2001 2005
2006 2010
2011 2015

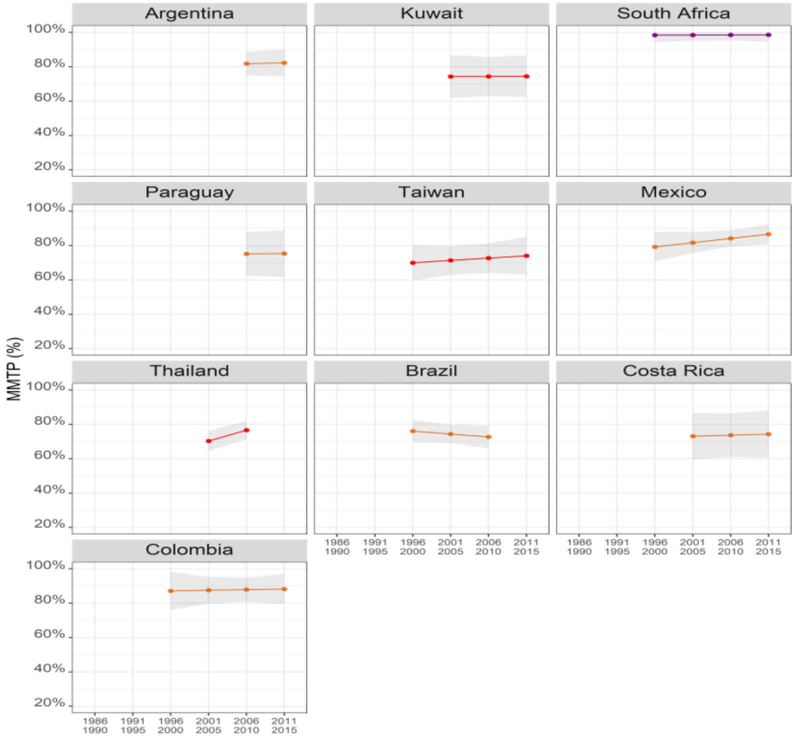
Argentina
Kuwait
South Africa
Paraguay
Taiwan
Mexico
Thailand
Brazil
Costa Rica
Colombia
MMTP (%)
100%
80%
60%
40%
20%
1986 1990
1991 1995
1996 2000
2001 2005
2006 2010
2011 2015

Figure S4. Temporal change in (A) MMT and (B) MMTP in the entire study population (top-left) and each of the 11 geographical regions from the sensitivity analysis using different range restrictions (1st to 99th)

(A)

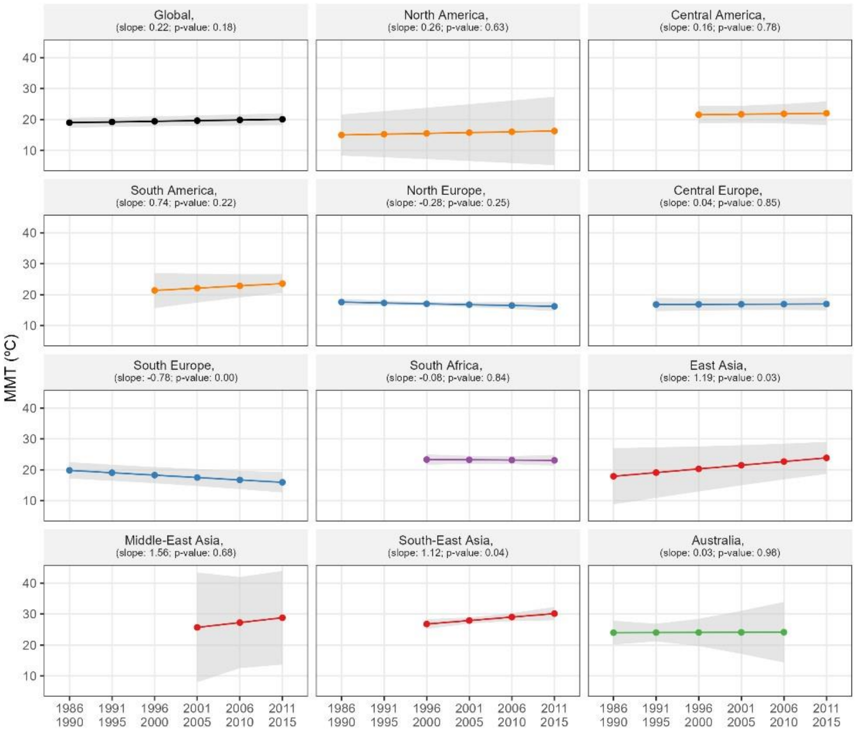

(B)

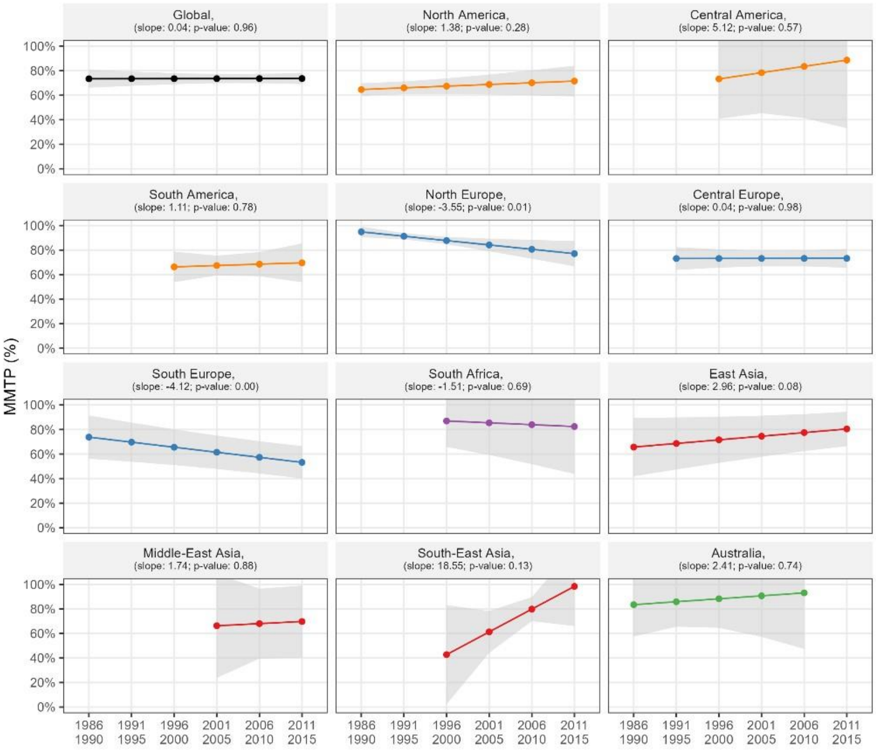

Figure S5. Distribution of the community-specific (A) MMT and (B) MMTP in the first (1986–1990) and last (2011–2015) subperiods in the entire population (top-left) and each of the 11 geographical regions from the sensitivity analysis using different range restrictions (1st to 99th)

(A)

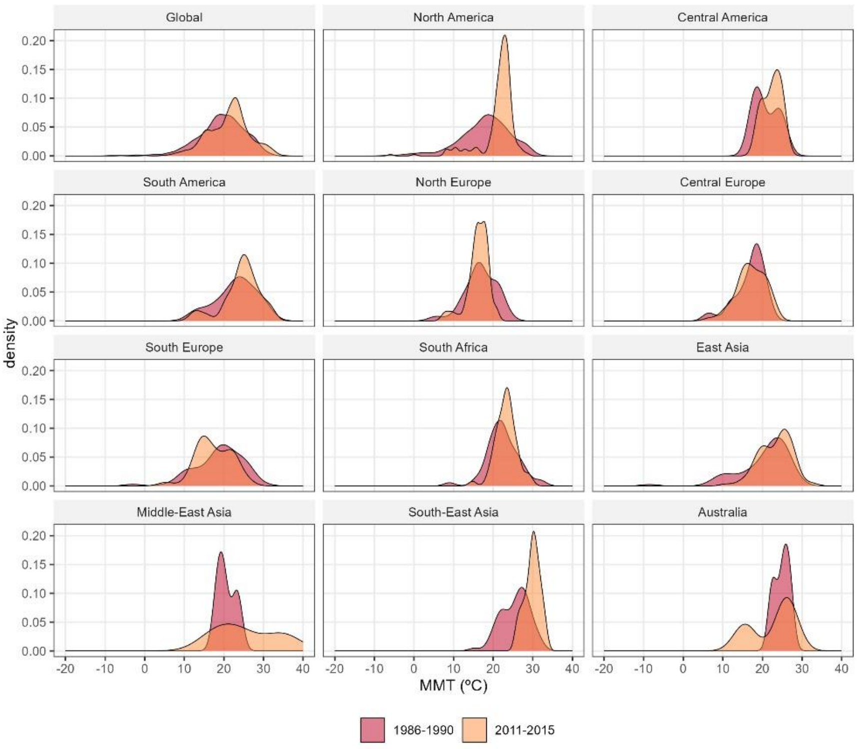

(B)

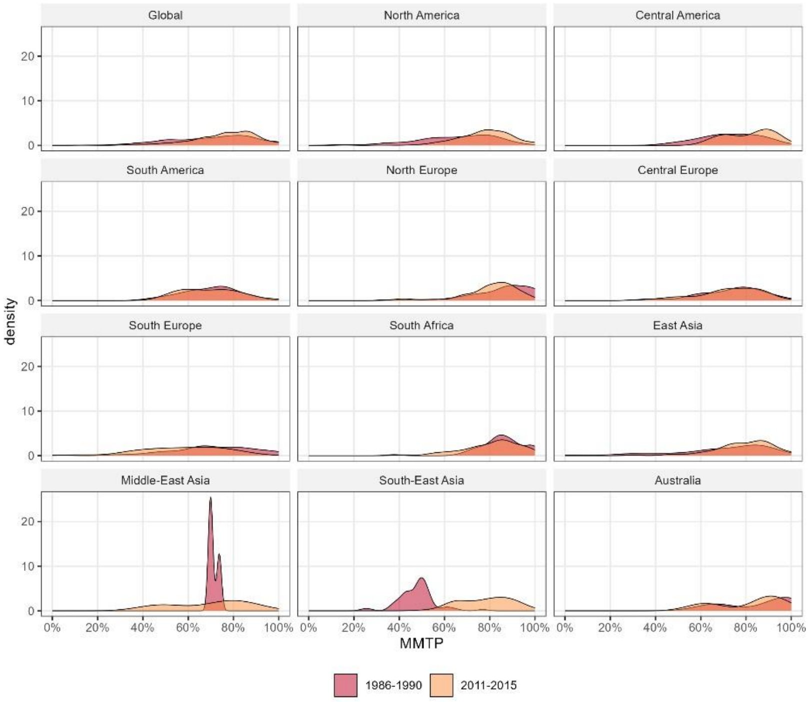

Figure S6. Temporal change in (A) MMT and (B) MMTP in the entire population (top-left) and each of the 11 geographical regions from the sensitivity analysis using a different study period from 2001 to 2015

(A)

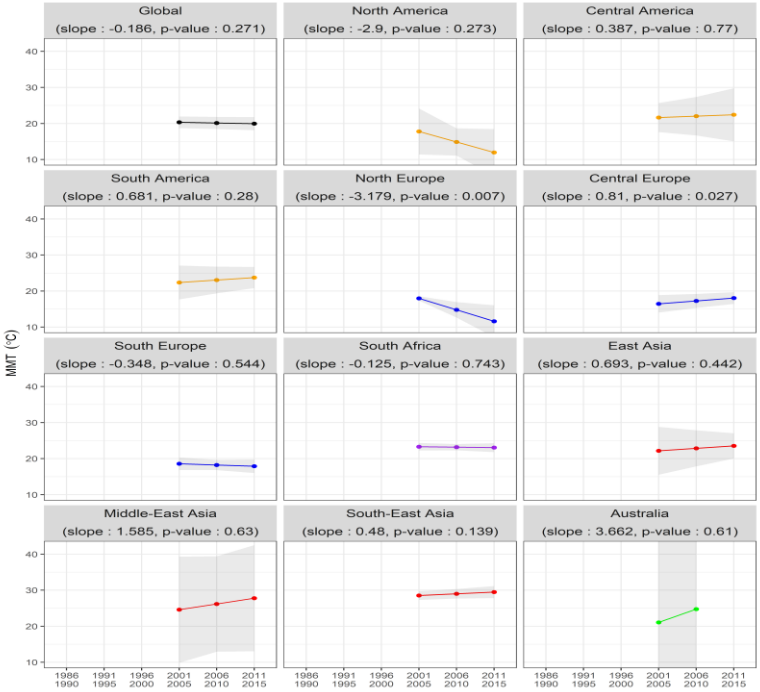

(B)

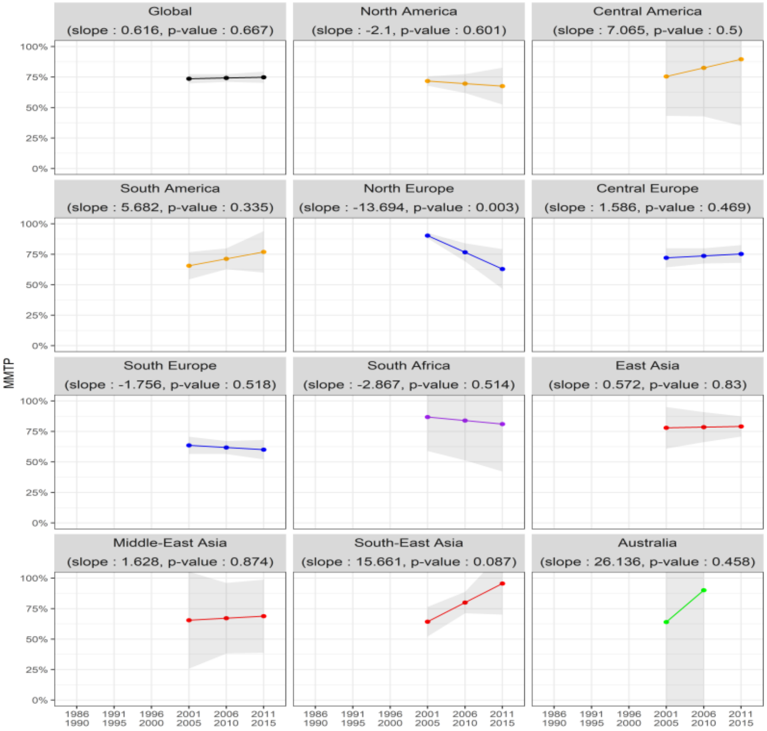

Figure S7. Temporal change in MMT (top) and MMTP (bottom) in each of the four climate zones from the sensitivity analysis using a different study period from 2001 to 2015

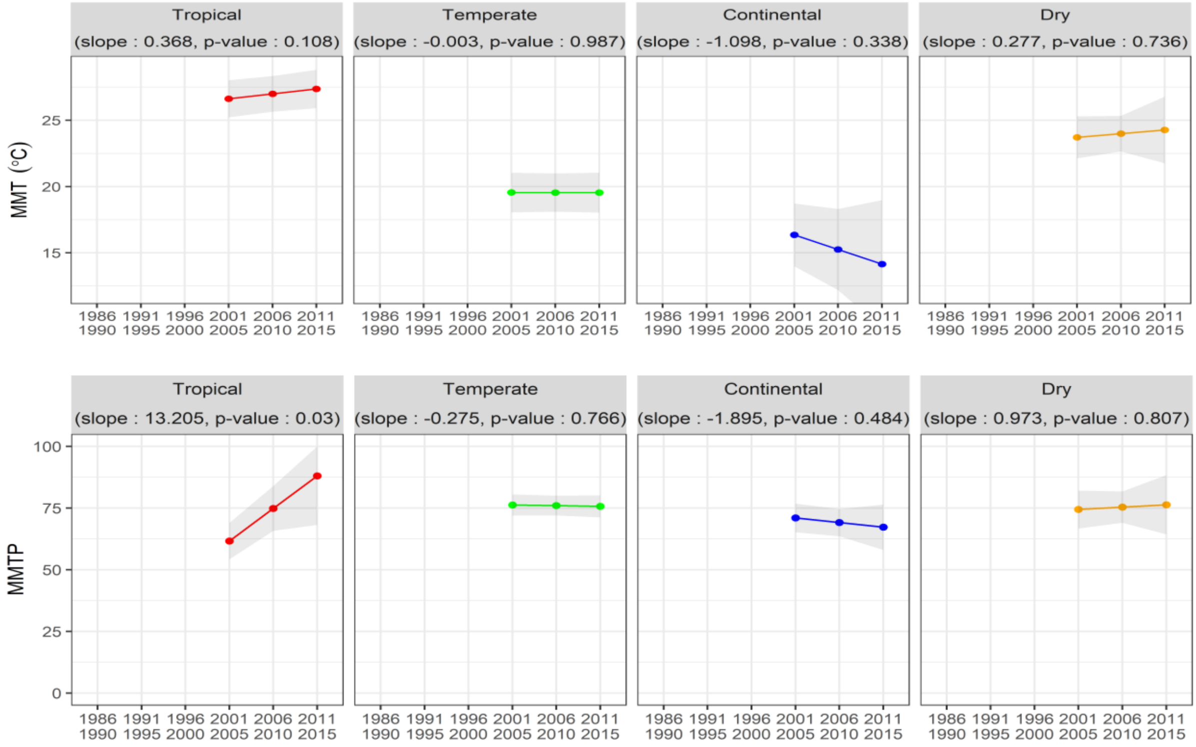

Supplement: Supplementary file 1 [file ee9-8-e334-s001.pdf]
